# Supplementary material for: Reliability, validity, and correlates of an AI voice emotion recognition app among nurses
Source: PLoS One. 2025 Dec 23;20(12):e0339365. doi: 10.1371/journal.pone.0339365 (PMC12725749; doi:10.1371/journal.pone.0339365)
Supplement: Attachment — Submitted filename: Review 2 PLOS.pdf [file pone.0339365.s002.pdf]

The growing burden of mental health issues among healthcare professionals represents a global challenge. Health systems exert significant structural pressures on professionals, including nurses. While there is often a tendency to focus solely on individual determinants, it is essential to consider environmental and systemic factors, particularly workplace-related determinants, that influence mental health. Recognizing this broader context allows for a more comprehensive understanding: addressing mental health issues requires not only individual-level support but also systemic changes to the environments that contribute to psychological distress. This holistic approach is a major contemporary challenge for improving health outcomes and could be a little bit more addressed in the discussion part of your work.
The study could benefit from numerous revisions to enhance methodological rigor and strengthen the discussion (while taking into account limitations) in order to effectively disseminate its findings.

#Abstract:

Objectives: "AI" should be fully written line 21 as it is the first time you introduce it.
Results: Describe what kind of "exercise" you are talking about. Is it a physical exercise, a professional exercise?
Conclusion: Line 36 the word "that" is useless.

#Introduction:

Line 54 you are referring to VER with a paper that you cite to highlight that the VER could be used in multiple fields such as screening for depression. When I read the paper you cited (4th reference), I could not find in the paper an application of VER except for Alzheimer's disease. I feel like this article was more about artificial intelligence techniques that could diagnose numerous diseases but not with the speech detection in general. The way you used that reference could be misleading and you should correct that.
Line 60, prefer the term "socio-demographic" rather than "personal".
Line 61 for your mention that "studies have found gradually increasing work pressure on nurses.", please provide references accordingly.
In line 66, since no evidence-based references support this statement, I would suggest using the term "could" instead of "can".
Line 68: Please rewrite the sentence. "developed based" is grammatically incorrect.

The introduction section is generally well structured but may be somewhat too concise. It could benefit from additional contextual information to provide a more comprehensive background on the topic.

#Methods:

Line 79: "study subjects" → "study participants"
Please indicate in this section how many researchers were recording the nurse's voice.
Please provide a translated questionnaire that nurses were answering.

Line 86: You mention that nurses recorded their voice for 60 seconds. However, in Figure 1 we can observe a length of conversation that appears to last 1 minutes and 30 seconds. Is 60 seconds a minimum required? Please clarify this. Also, could you please be more precise on what the nurse is supposed to talk about. No information is given on the content / topic of what the nurse is saying.
Line 91, 1st sentence: This should be included in the results section.
Line 91: Please make clearer the sentence for which you cite citation number 12 in which there is no data specifically on "emotion measurement tools" in this paper. Develop this aspect.
Ethical considerations section: please write it in past tense.
Line 109 "Research instruments": Please introduce this section properly.
Line 111: What was the rationale for collecting data on religion? Is there any scientific literature related to the study topic that justifies its inclusion? It is important to ensure that all collected data comply with international ethical standards, particularly regarding confidentiality and relevance.
Line 112: What do you mean by "living habits" or "work experience"? Please be more precise on the variables you collected. Indeed, it contrasts with the level of detail you provided for the scores in the research instruments section.
Line 197: Please specify how much data you tested the model with and provide more precise data on its results.
Line 207: You mention using t-tests. Detail the conditions under which this test was applied: was the normality of the variable distributions verified for this parametric test?

#Results

The results related to background characteristics should be compared in the discussion section with national statistics on nurses to support the external validity of your findings.
Table 1 (line 232): Could you clarify the choice of this title? The reference to "three groups" is unclear, and the use of the term "patient" seems inconsistent with your study population, which consists solely of healthcare professionals (nurses). Please provide a justification for this wording.
Tables: Please add in the note part (or title) the total number of participants included for these data. Keep a homogeneous formatting for this information across tables.
Line 254: Consider adding "in this study population" or "among nurses" to avoid overgeneralization of the findings.
Line 273: Please confirm the wording "negative automatic thoughts".

#Discussion

This section needs major revision.
While this section effectively presents the key results and relates them to existing literature, it lacks a critical analysis of the study. Notably, no limitations are discussed, despite several being apparent. For a more robust scientific discussion, a dedicated "Limitations" section should be added. You should consider addressing biases inherent to your study design, such as the small sample size, recall bias related to the use of self-administered questionnaires (especially for reporting physical symptoms or past medical history), and any other relevant methodological constraints. Clearly acknowledging these

limitations will strengthen the transparency and credibility of your findings. You should clearly state some limitations with a discussion of internal and external validity.

#General comments:

If the AI VER app detects and reports a high sadness score, are there specific referral procedures in place to connect the nurse with a mental health professional?

Additionally, your paper does not clearly explain how the various types of collected data were processed. Readers may be left with the impression that a significant amount of data was gathered without a clear account of how each type was analyzed or utilized. The clarity of your work would benefit from a more detailed explanation of your data collection methods and the subsequent data processing steps. This would enhance the transparency and reproducibility of your study.

Thank you for your work on that interesting and important topic!
