# Supplementary material for: Reliability, validity, and correlates of an AI voice emotion recognition app among nurses
Source: PLoS One. 2025 Dec 23;20(12):e0339365. doi: 10.1371/journal.pone.0339365 (PMC12725749; doi:10.1371/journal.pone.0339365)
Supplement: Attachment — Submitted filename: 20250613_Response Letter.docx [file pone.0339365.s004.docx]

***PLOS ONE***

Editor-in-Chief

Jun 13, 2025

Dear Editor-in-Chief,

Thank you very much for giving us the opportunity to again revise our paper entitled “Reliability and validity analysis of AI voice emotion recognition app and correlation between its results and the background variables and physical symptoms or diseases of nurses” (Manuscript ID PONE-D-25-20092). We have completed the required revisions, closely following the suggestions of the editor and reviewers. You may find the details of these revisions in the attached files. We believe that we have fulfilled all of the requirements for submission of the paper to *PLOS ONE*. If you find that any details have been overlooked, or if you need any further information, please do not hesitate to contact me.

Yours sincerely,

Wen-Pei Chang, RN, PhD

*PLOS ONE*

Referee Report on the Manuscript ID PONE-D-25-20092

“Reliability and validity analysis of AI voice emotion recognition app and correlation between its results and the background variables and physical symptoms or diseases of nurses”

We are grateful for your invaluable assistance in the revision of this paper. For this revision, we have closely followed the guidance provided by the editor and reviewers.

Detailed responses to each of the editor’s and reviewers’ comments have been provided below, with reviewers’ comments, followed by our comments and revisions.

In accordance with Editor’s suggestions:

1. Please ensure that your manuscript meets PLOS ONE's style requirements, including those for file naming. The PLOS ONE style templates can be found at https://journals.plos.org/plosone/s/file?id=wjVg/PLOSOne_formatting_sample_main_body.pdf and https://journals.plos.org/plosone/s/file?id=ba62/PLOSOne_formatting_sample_title_authors_affiliations.pdf

***Response:***

We thank the Editor for the reminder. We have ensured that our manuscript meets PLOS ONE’s style requirements, including those for file naming. We have referred to the official templates provided for the style examples. (see the Title page and Text)

2. Please note that PLOS ONE has specific guidelines on code sharing for submissions in which author-generated code underpins the findings in the manuscript. In these cases, we expect all author-generated code to be made available without restrictions upon publication of the work. Please review our guidelines at https://journals.plos.org/plosone/s/materials-and-software-sharing#loc-sharing-code and ensure that your code is shared in a way that follows best practice and facilitates reproducibility and reuse.

***Response:***

We understand PLOS ONE’s guidelines on code sharing for submissions involving author-generated code. However, the artificial intelligence (AI) voice emotion recognition (VER) algorithm used and developed in this study is legally under trade secret protection. Due to intellectual property and trade secret considerations, we are unable to disclose any details regarding the algorithm content, source code, or internal computational mechanisms. However, we confirm that the data analysis, results, and conclusions of this study are all based on the output of the validated VER system and do not involve the disclosure of or reliance on algorithm details. We are committed to adhering to relevant regulations and properly protecting proprietary technologies and trade secrets while ensuring the scientific rigor and reproducibility of the research.

3. Thank you for stating the following financial disclosure: [This research was funded through an industry-academia collaboration agreement between Shuang Ho Hospital, Taipei Medical University and Bamboo Technology Co., Ltd. in Taiwan, with the project code A-112-006-S.].

Please state what role the funders took in the study.  If the funders had no role, please state: ""The funders had no role in study design, data collection and analysis, decision to publish, or preparation of the manuscript.""

If this statement is not correct you must amend it as needed.

Please include this amended Role of Funder statement in your cover letter; we will change the online submission form on your behalf.

***Response:***

We thank the Editor for the reminder. Please update our information in the online submission form. We have also added the following paragraph to the cover letter: "This research was funded through an industry-academia collaboration agreement between Shuang Ho Hospital, Taipei Medical University, and Bamboo Technology Co., Ltd. in Taiwan, with the project code A-112-006-S. The funders had no role in the study design, data collection and analysis, decision to publish, or preparation of the manuscript. The first author, Chu-Ying Huang, is an employee of Bamboo Technology Co., Ltd. and contributed only to the conceptualization of the study. All other aspects of the research design, data collection and analysis, and manuscript preparation were solely carried out by the corresponding author, Wen-Pei Chang." (see the Cover letter)

4. Thank you for stating the following in the Acknowledgments Section of your manuscript: [This research was funded through an industry-academia collaboration agreement between Shuang Ho Hospital, Taipei Medical University and Bamboo Technology Co., Ltd. in Taiwan, with the project code A-112-006-S.]

We note that you have provided funding information that is not currently declared in your Funding Statement. However, funding information should not appear in the Acknowledgments section or other areas of your manuscript. We will only publish funding information present in the Funding Statement section of the online submission form.

Please remove any funding-related text from the manuscript and let us know how you would like to update your Funding Statement. Currently, your Funding Statement reads as follows: [This research was funded through an industry-academia collaboration agreement between Shuang Ho Hospital, Taipei Medical University and Bamboo Technology Co., Ltd. in Taiwan, with the project code A-112-006-S.].

Please include your amended statements within your cover letter; we will change the online submission form on your behalf.

***Response:***

We thank the Editor for this reminder. We understand that funding information should not appear in the Acknowledgments section and should only be placed in the Funding Statement section of the online submission form. We have removed all funding-related text from the manuscript. Please update our information in the online submission form. We have also added the following paragraph to the cover letter: "This research was funded through an industry-academia collaboration agreement between Shuang Ho Hospital, Taipei Medical University, and Bamboo Technology Co., Ltd. in Taiwan, with the project code A-112-006-S. The funders had no role in the study design, data collection and analysis, decision to publish, or preparation of the manuscript. The first author, Chu-Ying Huang, is an employee of Bamboo Technology Co., Ltd. and contributed only to the conceptualization of the study. All other aspects of the research design, data collection and analysis, and manuscript preparation were solely carried out by the corresponding author, Wen-Pei Chang." (see the Cover letter)

5. In the online submission form, you indicated that [The data that support the findings of this study are available on request from the corresponding author. The data are not publicly available due to privacy or ethical restrictions.].

All PLOS journals now require all data underlying the findings described in their manuscript to be freely available to other researchers, either 1. In a public repository, 2. Within the manuscript itself, or 3. Uploaded as supplementary information.

This policy applies to all data except where public deposition would breach compliance with the protocol approved by your research ethics board. If your data cannot be made publicly available for ethical or legal reasons (e.g., public availability would compromise patient privacy), please explain your reasons on resubmission and your exemption request will be escalated for approval.

***Response:***

We thank the Editor for this reminder. We understand PLOS ONE’s open data policy. In accordance with this policy, we have uploaded an Excel file of the raw data as supplementary information for other researchers to review and reproduce the study results. (see the S1 Dataset)

6. We note that Figure 1 in your submission contain copyrighted images. All PLOS content is published under the Creative Commons Attribution License (CC BY 4.0), which means that the manuscript, images, and Supporting Information files will be freely available online, and any third party is permitted to access, download, copy, distribute, and use these materials in any way, even commercially, with proper attribution. For more information, see our copyright guidelines: <http://journals.plos.org/plosone/s/licenses-and-copyright>.

We require you to either (1) present written permission from the copyright holder to publish these figures specifically under the CC BY 4.0 license, or (2) remove the figures from your submission:

1. You may seek permission from the original copyright holder of Figure 1 to publish the content specifically under the CC BY 4.0 license.

We recommend that you contact the original copyright holder with the Content Permission Form (http://journals.plos.org/plosone/s/file?id=7c09/content-permission-form.pdf) and the following text:

“I request permission for the open-access journal PLOS ONE to publish XXX under the Creative Commons Attribution License (CCAL) CC BY 4.0 (http://creativecommons.org/licenses/by/4.0/). Please be aware that this license allows unrestricted use and distribution, even commercially, by third parties. Please reply and provide explicit written permission to publish XXX under a CC BY license and complete the attached form.”

Please upload the completed Content Permission Form or other proof of granted permissions as an ""Other"" file with your submission.

In the figure caption of the copyrighted figure, please include the following text: “Reprinted from [ref] under a CC BY license, with permission from [name of publisher], original copyright [original copyright year].”

2. If you are unable to obtain permission from the original copyright holder to publish these figures under the CC BY 4.0 license or if the copyright holder’s requirements are incompatible with the CC BY 4.0 license, please either i) remove the figure or ii) supply a replacement figure that complies with the CC BY 4.0 license. Please check copyright information on all replacement figures and update the figure caption with source information. If applicable, please specify in the figure caption text when a figure is similar but not identical to the original image and is therefore for illustrative purposes only.

***Response:***

We thank the Editor for this reminder. We understand that all PLOS ONE content must be permitted for publication under the Creative Commons Attribution License (CC BY 4.0), and we have confirmed that the images in Fig 1 meet this requirement. We have sought permission from the original copyright holder of Fig 1 to publish the images in PLOS ONE under the CC BY 4.0 license, completed and uploaded the Content Permission Form, and added the following text to the caption of Fig 1: "(Image provided by Bamboo Technology Co., Ltd., published under a CC BY license, with permission from Bamboo Technology Co., Ltd., original copyright 2025.)" (see the Figure 1)

In accordance with Reviewer #1’s suggestions:

1. Ethical Considerations:

The manuscript mentions IRB approval and informed consent but lacks details on data anonymization and voice data protection. Given the sensitivity of biometric data, clarify how voice recordings were stored, processed, and anonymized to ensure participant confidentiality.

***Response:***

We thank the Reviewer for this reminder. We have added the following explanation to the Ethical considerations section: "To further protect the security of voice data and participant privacy, several data protection measures were implemented, including storing voice recordings on encrypted servers accessible only by authorized research team members using password protection. During data processing, all voice recordings were subjected to feature extraction using dedicated software, retaining only the acoustic parameters relevant to the analysis; the original voice files were not used or disclosed further. Any names or personally identifiable information contained in the recordings were deleted or anonymized prior to analysis, and the data were labeled with random codes; this completely removed any link to personal identities. All data were retained in accordance with the IRB-approved storage period and were securely destroyed after the retention period expired. The VER model used in the study was developed by Bamboo Technology Co., Ltd., which also funded the research. Although the company initiated the collaboration, it was not involved in data analysis, research processes, or decisions regarding study conclusions." (see the Ethical considerations Section on lines 126-138 on Page 8 and 9)

2. Sample Representation:

While the sample is predominantly female (85.4%), analyses involving menstrual cycles apply only to female participants. The manuscript should explicitly state whether male nurses were excluded from these specific analyses and discuss the implications for generalizability.

***Response:***

We thank the reviewer for the guidance. We have supplemented the following content in the Statistical analyses section: "As menstrual cycles are a physiological phenomenon unique to females, the menstrual cycle analysis was performed with only the data from female nurses. Male nurses were excluded from related analyses, and the results are only applicable to female nurses." (see the Statistical analyses Section on lines 280-283 on Page 16)

We have also added a note to the Menstrual cycles item in Table 2: "*Analysis only included the 298 female nurses." (see Table 2 on Page 26)

We have also added the following content to the Discussion section: "The analyses of specific physical symptoms (e.g., migraines and peptic ulcers) in this study included all of the participants (*N* = 349), that is, both male and female nurses. However, the analysis regarding menstrual cycles was limited to female nurses (*n* = 298); male participants were naturally excluded. As menstrual cycles are a female-specific physiological phenomenon, the findings cannot be directly generalized to male nurses or nurses of other genders. This analytical design limits the generalizability of the results across genders, particularly when exploring how physiological variables are related to emotions and physiological states. To enhance the external validity and applicability of future research, we recommend that future studies include participants of diverse genders or gender identities to examine the impact of their respective physiological and psychological variables on work-related emotions and health statuses, thus filling gaps in the existing body of research [46]." (see the Discussion Section on lines 440-450 on Page 36)

In response to this addition, we have cited the following study:

46. Polit FD, Beck CT. Nursing research: generating and assessing evidence for nursing practice. 11th Edition. Lippincott Williams & Wilkins; 2021.

3. Measurement Validity:

The AI VER app’s accuracy (70%) is moderate. Discuss limitations arising from this accuracy level (e.g., potential misclassification bias) and its impact on results.

Justify the choice of a decision tree classifier over more advanced models (e.g., neural networks) for emotion classification, addressing trade-offs between interpretability and performance.

***Response:***

We thank the reviewer for this reminder. We have supplemented the following content in the Research instruments section: "The preliminary model was trained using the RAVDESS data, which include 7,356 audio files of the voices of 24 professional actors (12 females and 12 males) between the ages of 21 and 40. All of these actors were native English speakers with North American accents. Their lines were delivered in various emotional states, which were then fine-tuned using a convolutional neural network classifier, achieving a preliminary accuracy of 79.2%." and "To enhance the appropriateness of the model for the expressions and culture of Mandarin speakers, the app collected voice data containing natural Mandarin spoken with a Taiwanese accent. The dataset contains 13,075 audio recordings submitted by 6,457 independent users and is 28,814 minutes in total. In the recordings, the participants talked freely and described their personal experiences and emotions. Three certified psychological counselors annotated all of the recordings using the same emotions used with RAVDESS. To process the subjectivity of the annotations, triangulation was adopted, and recordings with consistent annotations from at least two of the psychological counselors were retained. Ultimately, 1,961 training samples and 491 pen-and-paper test samples. The optimized Mandarin model has an accuracy rate of 79.8%. Note that due to the openness of the app, the dataset presents natural diversity but consequently lacks structured demographics, which prevents us from analyzing the performance of the model among certain ethnic groups." (see the Research instruments Section on lines 230-235 on Page 13 and 14, and lines 242-253 on Page 14 and 15)

We have also added the following content to the Discussion section: "The accuracy of the AI VER app used in this study for emotion recognition was approximately 70%, which is deemed moderate. This may indicate potential misclassification bias, thereby limiting the accuracy and explanatory power of the results. Researchers could consider in future studies using comparison approaches with greater performance or multiple models to validate and enhance the reliability of emotion recognition [49]. Furthermore, regarding the choice of emotion classification model, this study employed a decision tree classifier, primarily due to its strong interpretability and intuitive classification logic, which can help clinical practitioners to better understand classification rules. Although more advanced models such as neural networks may offer higher prediction performance, their complexity could reduce interpretability and acceptance in practical applications [50]. Therefore, the decision tree model in this study provided a reasonable balance between performance and interpretability." (see the Discussion section on lines 464-475 on Page 37 and 38)

In response to this addition, we have cited the following studies:

49. Shatte ABR, Hutchinson DM, Teague SJ. Machine learning in mental health: a scoping review of methods and applications. Psychol Med. 2019;49:1426–1448. doi:10.1017/S0033291719000151

50. Lopes B, Soares LS, Gonçalves MA, Prates RO. A human-centered multiperspective and interactive visual tool for explainable machine learning. JBCS. 2025;31:11–35. doi:10.5753/jbcs.2025.3982

4. Causality vs. Correlation:

The discussion occasionally implies causation (e.g., fear leading to peptic ulcers). Clarify that the study identifies associations, not causality, and avoid overinterpretation of mechanisms without longitudinal or experimental evidence.

***Response:***

We thank the reviewer for this reminder. We have reorganized the content of the Discussion as follows: "Although Lee et al. noted that fear may be associated with increased gastric acid secretion and weakened gastric mucosal protection mechanisms and is thereby linked to the risk of peptic ulcers [44], the cross-sectional design of this study could only identify correlations and thus could not confirm that fear directly causes peptic ulcers. This study also revealed that lower happiness scores and higher sadness scores were significantly correlated with irregular menstrual cycles in female nurses. Existing literature suggests that emotional stress may disrupt the normal functioning of the hypothalamic-pituitary-ovarian axis, thereby disturbing the balance among luteinizing hormone, follicle-stimulating hormone, estrogen, and progesterone. Changes in these hormones may be related to menstrual cycle irregularities [45]. However, due to the cross-sectional design of this study, we similarly could not confirm that emotional states directly cause menstrual cycle irregularities. Longitudinal or experimental studies are needed to further explore potential mechanisms and causal relationships." (see the Discussion Section on lines 427-439 on Page 35 and 36)

5. Confounding Variables:

Key confounders like workload intensity, job-specific stressors, or comorbid mental health conditions (e.g., anxiety) are not controlled. Acknowledge this limitation and suggest future studies to account for these variables.

***Response:***

We thank the reviewer for this reminder. We have added the following content to the Limitations section: "We did not control confounders such as workload intensity, work stress sources, or mental health conditions (e.g., anxiety and insomnia) in this study. These uncontrolled variables may have affected the findings. Past studies have indicated that work environments and psychological stress are significantly associated with the emotions and health status of nurses [55, 56]. Therefore, we recommend that future studies incorporate these key variables into analytical models to enhance the internal validity and explanatory power of the findings and to provide a more comprehensive understanding of the complex relationship between the emotions and health of nurses." (see the Limitations Section on lines 498-505 on Page 39)

In response to this addition, we have cited the following studies:

55. Pereira MC, Eberhardt LD, de Carvalho M. Working conditions and mental illness among nursing workers. Rev Bras Med Trab. 2024;22:e2022980. doi:10.47626/1679-4435-2022-980

56. Babapour AR, Gahassab-Mozaffari N, Fathnezhad-Kazemi A. Nurses' job stress and its impact on quality of life and caring behaviors: a cross-sectional study. BMC Nurs. 2022;21:75. doi:10.1186/s12912-022-00852-y

6. Test-Rest Reliability:

The retest sample size (n=30) is small. While a 15% loss rate is mentioned, consider expanding the retest cohort to enhance reliability estimates, particularly for fear (moderate ICC).

***Response:***

We thank the reviewer for this reminder. We have added the following content to the Discussion section: "However, the retest sample size in this study was small, and the ICC of fear, in particular, only indicated moderate correlation; therefore, the stability and precision could be somewhat limited [54]." (see the Discussion Section on lines 483-485 on Page 38)

In response to this addition, we have cited the following study:

54. Mokkink LB, de Vet H, Diemeer S, Eekhout I. Sample size recommendations for studies on reliability and measurement error: an online application based on simulation studies. Health Serv Outcomes Res Method. 2023;23:241–265. https://doi.org/10.1007/s10742-022-00293-9

7. Cultural Context:

The study was conducted in Taiwan. Discuss potential cultural influences on emotion expression (e.g., social desirability bias) and how these might affect the app’s performance or generalizability to other populations.

***Response:***

We thank the reviewer for this reminder. We have added the following content to the Limitations section: "This study was conducted in Taiwan; as such, the results could have been influenced by the local cultural context because individuals in Asian cultures often suppress or internalize negative emotions to meet social expectations and maintain interpersonal harmony. This may have led to social desirability bias, potentially affecting the accuracy of self-reported data and the emotion recognition results of the app [57]. Additionally, significant cultural differences in attitudes and approaches to emotional expression and interpretation may limit the generalizability of our findings and the applicability of the app to other cultural groups [58]. We therefore suggest that future studies include cross-cultural samples to validate the effectiveness and stability of this tool across diverse cultural contexts and to further enhance the external validity and practical value of the study results." (see the Discussion Section on lines 506-515 on Page 39 and 40)

In response to this addition, we have cited the following studies:

57. Kim J, Stead GB. Validation of Korean version of the anger discomfort scale. Int J Adv Counselling. 2024;46:679–701. doi:10.1007/s10447-024-09547-2

58. Deng X, An S, Cheng C. Cultural differences in the implicit and explicit attitudes toward emotion regulation. Pers Individ Dif. 2019;149:220–222. doi:10.1016/j.paid.2019.05.057

8. Data Availability:

PLOS ONE mandates public data availability unless restricted by ethical/legal concerns. The current statement (“available upon request”) may not comply. Either deposit data in a repository with access controls or provide a detailed justification for restrictions.

***Response:***

We thank the reviewer for this reminder. We understand PLOS ONE’s open data policy. In accordance with this policy, we have uploaded an Excel file of the raw data as supplementary information for other researchers to review and reproduce the study results. (see the S1 Dataset)

9. Figures and Tables:

Figure 1 is referenced but not included in the manuscript draft. Ensure all figures are properly formatted and captioned.

Table 1’s title (“three patient groups”) is inaccurate (participants are nurses). Revise for clarity.

***Response:***

We thank the reviewer for this guidance and assistance. We have placed the caption of Fig 1 directly after the paragraph where it is first referenced and ensured that all Fig 1 files are properly formatted (Fig1.tif). (see the Research design Section on lines 115-116 on Page 7 and Fig 1)

Furthermore, we thank you for the reminder regarding the title of Table 1, which we have corrected to "Correlation between personal background variables and emotion recognition results of app (*N*=349)." (see Table 1 on Page 19)

10. Language and Clarity:

Several sentences are grammatically awkward or unclear (e.g., “The scores of the four emotions are A (anger), B (fear), C (happiness), and D (sadness)…”). Professional editing for language fluency is recommended.

***Response:***

We thank the reviewer for this valuable suggestion. We have revised all unclear or grammatically awkward sentences in the manuscript. For instance, we have corrected the aforementioned sentence to "The four emotion scores are denoted as A (anger), B (fear), C (happiness), and D (sadness), with the condition that A + B + C + D = 1." (see the Research instruments Section on lines 257-258 on Page 15)

We have engaged a professional English editing service to thoroughly revise and refine the language, thereby improving both clarity and fluency. A certificate of editing has been included.

11. Conflict of Interest:

The VER model was developed by Bamboo Technology Co., Ltd., which funded the study. While the authors declare no competing interests, explicitly address potential biases (e.g., whether the company influenced data analysis/interpretation).

***Response:***

We thank the Reviewer for this reminder. We have added the following explanation to the Ethical considerations section: "The VER model used in the study was developed by Bamboo Technology Co., Ltd., which also funded the research. Although the company initiated the collaboration, it was not involved in data analysis, research processes, or decisions regarding study conclusions." (see the Ethical considerations Section on lines 135-138 on Page 8 and 9)

12. Methodological Rigor:

The app’s training data (RAVDESS and “members”) lacks specificity. Describe the demographic composition of training datasets to assess model generalizability.

***Response:***

We thank the reviewer for this reminder. We have supplemented the following content in the Research instruments section: "The preliminary model was trained using the RAVDESS data, which include 7,356 audio files of the voices of 24 professional actors (12 females and 12 males) between the ages of 21 and 40. All of these actors were native English speakers with North American accents. Their lines were delivered in various emotional states, which were then fine-tuned using a convolutional neural network classifier, achieving a preliminary accuracy of 79.2%." and "To enhance the appropriateness of the model for the expressions and culture of Mandarin speakers, the app collected voice data containing natural Mandarin spoken with a Taiwanese accent. The dataset contains 13,075 audio recordings submitted by 6,457 independent users and is 28,814 minutes in total. In the recordings, the participants talked freely and described their personal experiences and emotions. Three certified psychological counselors annotated all of the recordings using the same emotions used with RAVDESS. To process the subjectivity of the annotations, triangulation was adopted, and recordings with consistent annotations from at least two of the psychological counselors were retained. Ultimately, 1,961 training samples and 491 pen-and-paper test samples. The optimized Mandarin model has an accuracy rate of 79.8%. Note that due to the openness of the app, the dataset presents natural diversity but consequently lacks structured demographics, which prevents us from analyzing the performance of the model among certain ethnic groups." (see the Research instruments Section on lines 230-235 on Page 13 and Page 14, and lines 242-253 on Page 14 and 15)

13. Methodological Rigor:

For menstrual cycle assessment, clarify how “irregularity” was operationalized (self-report vs. clinical criteria) to ensure validity.

***Response:***

We thank the reviewer for this correction. We have supplemented the operational definition of menstrual cycle irregularity in the Research instruments section and clarified that the data were self-reported rather than based on clinical criteria to ensure the validity and transparency of the research instruments. The relevant content is as follows: "For menstrual cycle regularity assessment, we designed a short questionnaire containing seven question items [23], including two questions regarding menstrual cycle length (“How long is your menstrual cycle usually?” and “Have you experienced any apparent changes to your menstrual cycle within the past six months?”), two questions regarding menstruation periods (“How long does your menstruation period usually last? Has it ever been longer than 35 days or shorter than 24 days or changed significantly within the past six months?”), two questions regarding menstrual flow (“How much is your menstrual flow usually?” and “Have you experienced any apparent changes to your menstrual flow within the past six months?”), and one question regarding menstruation abnormalities (“Have you experienced extreme pain during your menstruation period, abnormal bleeding in between menstruation periods, continuous spotting during menstruation periods, or cessation of menstruation during the past six months?”). Any response indicating abnormalities to these questions was regarded as menstrual irregularity. Five experts with wide experience in related fields (including attending gynecologists and professors of gynecological nursing) assessed the content validity of each question on a scale from 1 to 4 (1 indicating complete inappropriateness and 4 indicating complete appropriateness) based on appropriateness and relevance. The resulting content validity index (CVI) was 94%." (see the Research instruments Section on lines 155-172 on Page 9 and 10)

In accordance with Reviewer #2’s suggestions:

1. Abstract:
Objectives: “AI” should be fully written line 21 as it is the first time you introduce it.
Results: Describe what kind of “exercise” you are talking about. Is it a physical exercise, a professional exercise?
Conclusion: Line 36 the word “that” is useless.

***Response:***

We thank the reviewer for the careful review and suggestions. We have fully written "AI" as "artificial intelligence" in the Objectives, where it is first introduced. In the Results, we have clarified that "exercise" means "physical exercise". In the Conclusion section, we have removed the redundant "that" to improve fluency. (see the Abstract on Page 2)

2. Introduction:
Line 54 you are referring to VER with a paper that you cite to highlight that the VER could be used in multiple fields such as screening for depression. When I read the paper you cited (4th reference), I could not find in the paper an application of VER except for Alzheimer’s disease. I feel like this article was more about artificial intelligence techniques that could diagnose numerous diseases but not with the speech detection in general. The way you used that reference could be misleading and you should correct that.

***Response:***

We thank the reviewer for this reminder. We have added three relevant citations to the text to strengthen our discourse and ensure consistency between the content and our citations. (see the Introduction Section on lines 58 on Page 4)

In response to this addition, we have cited the following studies:

6. Kim D, Yi B, Won Y. Speech emotion recognition in people at high risk of dementia. Dement Neurocogn Disord. 2024;23:146–160. doi:10.12779/dnd.2024.23.3.146

7. Hansen L, Zhang YP, Wolf D, Sechidis K, Ladegaard N, Fusaroli R. A generalizable speech emotion recognition model reveals depression and remission. Acta Psychiatr Scand. 2022;145:186–199. doi:10.1111/acps.13388

8. Zhao X, Wen H, Xu G, et al. Validity, feasibility, and effectiveness of a voice-recognition based digital cognitive screener for dementia and mild cognitive impairment in community-dwelling older Chinese adults: a large-scale implementation study. Alzheimers Dement. 2024;20:2384–2396. doi:10.1002/alz.13668

3. Introduction:
Line 60, prefer the term “socio-demographic” rather than “personal”.

***Response:***

We thank the reviewer for this reminder. We have changed "personal" to the more precise "socio-demographic" for better accuracy and professionalism in the terminology. (see the Introduction Section on lines 62 on Page 5)

4. Introduction:

Line 61 for your mention that “studies have found gradually increasing work pressure on nurses.”, please provide references accordingly.

***Response:***

We thank the reviewer for this reminder. We have added the appropriate citations to the end of this sentence. (see the Introduction Section on lines 64 on Page 5)

In response to this addition, we have cited the following studies:

11. Al-Yaqoubi S, Arulappan J. Sources of work-related stress among omani nurses: a cross-sectional study. J Patient Exp. 2023;10:23743735231166504. doi:10.1177/23743735231166504

12. Gmayinaam VU, Nortey AN, Sedode S, et al. Work-related stress among nurses: a comparative cross-sectional study of two government hospitals in Ghana. BMC Public Health. 2024;24:2257. doi:10.1186/s12889-024-19757-3

5. Introduction:

In line 66, since no evidence-based references support this statement, I would suggest using the term “could” instead of “can”.

***Response:***

We thank the reviewer for these valuable suggestions. The statement here indeed lacks support from empirical literature so far. Thus, we have changed "can" to "could" to convey a more cautious tone. (see the Introduction Section on lines 72 on Page 5)

6. Introduction:

Line 68: Please rewrite the sentence. “developed based” is grammatically incorrect.
***Response:***

We thank the reviewer for this correction. We have corrected this sentence as follows: "AI-powered emotional recognition apps can automatically detect and analyze users’ emotional states." (see the Introduction Section on lines 74-75 on Page 5)

7. Introduction:

The introduction section is generally well structured but may be somewhat too concise. It could benefit from additional contextual information to provide a more comprehensive background on the topic.

***Response:***

We thank the reviewer for this suggestion. We have supplemented more background- and context-related information in the Introduction to provide a more complete introduction to the topic and research background as well as to elucidate the motivation and importance of this study. (see the Introduction Section on lines 43-84 on Page 4-6)

8. Methods:
Line 79: “study subjects”  “study participants”

***Response:***

We thank the reviewer for this suggestion. We have changed "study subjects" to "study participants" to stress the active role of the participants in this study and our ethical respect for them. (see the Research design Section on lines 88 on Page 6)

9. Methods:
Please indicate in this section how many researchers were recording the nurse’s voice.

***Response:***

We thank the reviewer for this suggestion. We have clarified the number of researchers that took part in recording the nurses’ voices in this section to enhance the clarity and transparency of the methods, as follows: "The voice recordings and questionnaires from the participants were all collected by a single research nurse to ensure the consistency and reliability of the data." (see the Research design Section on lines 88-90 on Page 6)

10. Methods:
Please provide a translated questionnaire that nurses were answering.

***Response:***

As suggested by the reviewer, we have provided a complete translation of the questionnaire that the nurses filled out in the attachments for review and content confirmation. (see the translated questionnaire)

11. Methods:
Line 86: You mention that nurses recorded their voice for 60 seconds. However, in Figure 1 we can observe a length of conversation that appears to last 1 minutes and 30 seconds. Is 60 seconds a minimum required? Please clarify this. Also, could you please be more precise on what the nurse is supposed to talk about. No information is given on the content / topic of what the nurse is saying.

***Response:***

We thank the reviewer for this correction. We have added details regarding the length of the recordings as follows: "To collect adequate voice data for analysis, each nurse was required to record at least 60 seconds of their voice. Note that no upper limit was given to the recordings; therefore, some of the recordings were over 60 seconds long. The recording segment shown in Fig 1, for instance, was 1 minute and 30 seconds long. To ensure voice data authenticity and diversity in emotional expression and the recorded content, we asked the nurses to describe their work experiences and emotions regarding the past day in a natural tone or share their challenges, feelings, and realizations in clinical work." (see the Research design Section on lines 96-103 on Page 6 and 7)

12. Methods:
Line 91, 1st sentence: This should be included in the results section.

***Response:***

We thank the reviewer for this correction. We have moved the sentence, "Information was collected from a total of 349 nurses.", to the results as suggested for better article structure and logic. (see the Results Section on lines 301 on Page 17)

13. Methods:

Line 91: Please make clearer the sentence for which you cite citation number 12 in which there is no data specifically on “emotion measurement tools” in this paper. Develop this aspect.

***Response:***

We thank the reviewer for this correction. We have changed the paper originally cited to a more appropriate paper that is directly associated with emotion measurement tools to enhance the accuracy and consistency of the discourse. (see the Research design Section on lines 108-109 on Page 7)

In response to this addition, we have cited the following studies:

20. Carlozzi NE, Kallen MA, Brickell TA, et al. Measuring emotional suppression in caregivers of adults with traumatic brain injury. Rehabil Psychol. 2020;65:455–470. doi:10.1037/rep0000291

21. Grant M, Salsman NL, Berking M. The assessment of successful emotion regulation skills use: development and validation of an English version of the Emotion Regulation Skills Questionnaire. PLoS One. 2018;13:e0205095. doi:10.1371/journal.pone.0205095

14. Methods:

Ethical considerations section: please write it in past tense.
***Response:***

We thank the reviewer for this correction. We have changed the Ethical considerations section to past tense in accordance with writing standards as follows: "This study was approved by the Joint Institutional Review Board of Taipei Medical University (approval number: N202309075) prior to data collection and analysis. Before data collection, the researchers explained the purpose and procedures of the study in detail to the participating nurses and then obtained their written informed consent. During the study period, nurses were free to withdraw or discontinue participation at any time without affecting their professional rights or responsibilities. All data provided by the nurses were used solely for academic research purposes and were de-identified to ensure privacy and confidentiality." (see the Ethical considerations Section on lines 119-125 on Page 8)

15. Methods:

Line 109 “Research instruments”: Please introduce this section properly.

***Response:***

We thank the reviewer. We have added an opening statement to the Research instruments section as follows: "The data collection tools used in this study included the basic data and physical symptoms or diseases of the nurses, the Positive and Negative Affect Schedule (PANAS), the Oxford Happiness Questionnaire (OHQ), the Perceived Stress Scale (PSS), the Beck Depression Inventory-II (BDI-II), the Novaco Anger Inventory-Short Form (NAI-25), and our AI VER app. The details are as follows:" (see the Research instruments Section on lines 141-145 on Page 9)

16. Methods:

Line 111: What was the rationale for collecting data on religion? Is there any scientific literature related to the study topic that justifies its inclusion? It is important to ensure that all collected data comply with international ethical standards, particularly regarding confidentiality and relevance.

***Response:***

We thank the reviewer for this correction. We have added our reason for collecting religion data in this study as follows: "Note that religious beliefs have been shown to play a crucial role in an individual’s emotions and mental health [13]. As nurses work in high-stress environments, religious beliefs may serve as a protective factor that regulates negative emotions [14]." (see the Introduction Section on lines 64-67 on Page 5)

In response to this addition, we have cited the following studies:

13. Lucchetti G, Koenig HG, Lucchetti ALG. Spirituality, religiousness, and mental health: A review of the current scientific evidence. World J Clin Cases. 2021;9:7620–7631. doi:10.12998/wjcc.v9.i26.7620

14. Harris S, Tao H. The impact of US nurses' personal religious and spiritual beliefs on their mental well-being and burnout: a path analysis. J Relig Health. 2022;61:1772–1791. doi:10.1007/s10943-021-01203-y

Furthermore, we again confirm that all of the collected data meet international ethical standards, especially in terms of information confidentiality and research relevance. The relevant content is as follows: "During the study period, nurses were free to withdraw or discontinue participation at any time without affecting their professional rights or responsibilities. All data provided by the nurses were used solely for academic research purposes and were de-identified to ensure privacy and confidentiality."(see the Ethical considerations Section on lines 122-125 on Page 8)

17. Methods:

Line 112: What do you mean by “living habits” or “work experience”? Please be more precise on the variables you collected. Indeed, it contrasts with the level of detail you provided for the scores in the research instruments section.

***Response:***

We thank the reviewer for this correction. We have changed "work experience" to "years of work experience" and added "(including whether they smoked, consumed alcohol or coffee, or exercised)" after "living habits". (see the Research instruments Section on lines 147-149 on Page 9)

18. Methods:

Line 197: Please specify how much data you tested the model with and provide more precise data on its results.

***Response:***

We thank the reviewer for this reminder. We have supplemented an explanation of the amount of data used to test the model in the Research instruments section and provided test result data to enhance the transparency and explanatory power of the results. The relevant content is as follows: "The preliminary model was trained using the RAVDESS data, which include 7,356 audio files of the voices of 24 professional actors (12 females and 12 males) between the ages of 21 and 40. All of these actors were native English speakers with North American accents. Their lines were delivered in various emotional states, which were then fine-tuned using a convolutional neural network classifier, achieving a preliminary accuracy of 79.2%." and "To enhance the appropriateness of the model for the expressions and culture of Mandarin speakers, the app collected voice data containing natural Mandarin spoken with a Taiwanese accent. The dataset contains 13,075 audio recordings submitted by 6,457 independent users and is 28,814 minutes in total. In the recordings, the participants talked freely and described their personal experiences and emotions. Three certified psychological counselors annotated all of the recordings using the same emotions used with RAVDESS. To process the subjectivity of the annotations, triangulation was adopted, and recordings with consistent annotations from at least two of the psychological counselors were retained. Ultimately, 1,961 training samples and 491 pen-and-paper test samples. The optimized Mandarin model has an accuracy rate of 79.8%. Note that due to the openness of the app, the dataset presents natural diversity but consequently lacks structured demographics, which prevents us from analyzing the performance of the model among certain ethnic groups." (see the Research instruments Section on lines 230-235 on Page 13 and 14, and lines 242-253 on Page 14 and 15)

19. Methods:

Line 207: You mention using *t*-tests. Detail the conditions under which this test was applied: was the normality of the variable distributions verified for this parametric test?
***Response:***

We thank the reviewer for this correction. We have supplemented the following content in the Statistical analyses section: "Before conducting t-tests and one-way ANOVAs, we first used the Shapiro-Wilk test to confirm whether the data groups followed normal distributions. If there were non-normal distributions, the Mann-Whitney U test or Kruskal-Wallis test was used instead." (see the Statistical analyses Section on lines 277-280 on Page 16)

20. Results
The results related to background characteristics should be compared in the discussion section with national statistics on nurses to support the external validity of your findings.

***Response:***

We thank the reviewer for this valuable suggestion. We have added a comparison of the background characteristics of the participants in this study with national statistics on nurses to the Discussion as follows: "According to a nursing workforce survey conducted by the Taiwan Union of Nurses Association in December 2024, the mean age of nurses in Taiwan is 39, and 95.4% of the nurses are women [38]. The largest age group in the sample of this study was between 25 and 29 years old. We limited the participants to those between the ages of 20 and 45 and excluded those who were pregnant or taking hormonal drugs. As a result, the sample distributions were relatively young and mostly female. This differs from the age structure of the national nursing workforce, which may impact the external validity of the study results. We also eliminated potential confounding factors affecting physiological and psychological variables when designing this study; as a consequence, the samples cannot fully represent the entire national nursing workforce. Therefore, we suggest that future studies include nurses of different age groups with different physiological conditions to enhance the generalizability of the findings." (see the Discussion Section on lines 387-397 on Page 33 and 34)

21. Results

Table 1 (line 232): Could you clarify the choice of this title? The reference to “three groups” is unclear, and the use of the term “patient” seems inconsistent with your study population, which consists solely of healthcare professionals (nurses). Please provide a justification for this wording.

***Response:***

We thank the reviewer for this correction. We have corrected the title of Table 1 to "Table 1. Correlation between personal background variables and emotion recognition results of app (*N*=349)." (see Table 1 on lines 310 on Page 19)

22. Results

Tables: Please add in the note part (or title) the total number of participants included for these data. Keep a homogeneous formatting for this information across tables.

***Response:***

We thank the reviewer for this suggestion. We have added the total number of participants included in the data in the title or notes of all of the tables. (see Table 1-Table 4)

23. Results

Line 254: Consider adding “in this study population” or “among nurses” to avoid overgeneralization of the findings.

***Response:***

We thank the reviewer for this suggestion. We have revised the content of this sentence as follows: "These findings suggest that in the studied sample of nurses, irregular menstrual cycles were associated with reduced positive emotions and increased negative emotions." (see the Results Section on lines 332-334 on Page 24)

24. Results

Line 273: Please confirm the wording “negative automatic thoughts”.
***Response:***

We thank the reviewer for this suggestion. We have revised the content of this sentence as follows: "anger showed a significant positive correlation with the total NAI score (*r* = .420, *p* < .001); happiness showed a significant negative correlation with the total NAI score (*r* = -.154, *p* = .004); and sadness also showed a significant negative correlation with the total NAI score (*r* = -.158, *p* = .003). These findings suggest that among the nurses in this study, the recognition of anger, happiness, and sadness in the app reflected the measured outcomes of the NAI total score." (see the Results Section on lines 353-359 on Page 27 and 28)

25. Discussion
This section needs major revision.
While this section effectively presents the key results and relates them to existing literature, it lacks a critical analysis of the study. Notably, no limitations are discussed, despite several being apparent. For a more robust scientific discussion, a dedicated “Limitations” section should be added. You should consider addressing biases inherent to your study design, such as the small sample size, recall bias related to the use of self-administered questionnaires (especially for reporting physical symptoms or past medical history), and any other relevant methodological constraints. Clearly acknowledging these limitations will strengthen the transparency and credibility of your findings. You should clearly state some limitations with a discussion of internal and external validity.
***Response:***

We thank the reviewer for this valuable suggestion. We have made major revisions to the Discussion and added details to the Limitations section to explain the potential limitations of this study. (see the Discussion and Limitations Section on lines 379-515 on Page 33-40)

26. General comments:
If the AI VER app detects and reports a high sadness score, are there specific referral procedures in place to connect the nurse with a mental health professional?
***Response:***

We thank the reviewer for mentioning this. This study focused on validating the emotion recognition performance and feasibility of the AI VER app. No concrete referral or subsequent intervention processes have been designed for when higher sadness scores are detected. We have therefore added the following explanation to the Conclusion: "This study primarily focused on the measurement validity and correlation analysis of the AI VER app in the nursing population without incorporating clinical referral or mental health intervention processes. In other words, although the system can detect higher sadness scores, it was used solely for data collection and analysis in this stage of research. No automated or real-time mental health referral mechanisms were designed. Future applications of such technologies must give careful consideration to ethical responsibilities and be integrated with in-hospital mental health resources or workplace counseling mechanisms to ensure timely professional support and intervention when high-risk emotions are detected." (see the Conclusion Section on lines 523-530 on Page 40 and 41)

27. Additionally, your paper does not clearly explain how the various types of collected data were processed. Readers may be left with the impression that a significant amount of data was gathered without a clear account of how each type was analyzed or utilized. The clarity of your work would benefit from a more detailed explanation of your data collection methods and the subsequent data processing steps. This would enhance the transparency and reproducibility of your study.
***Response:***

We thank the reviewer for this valuable suggestion. We have added explanations regarding the data collection and subsequent data processing procedures to the part describing the AI VER app in the Research instruments section as follows: "The preliminary model was trained using the RAVDESS data, which include 7,356 audio files of the voices of 24 professional actors (12 females and 12 males) between the ages of 21 and 40. All of these actors were native English speakers with North American accents. Their lines were delivered in various emotional states, which were then fine-tuned using a convolutional neural network classifier, achieving a preliminary accuracy of 79.2%." and "To enhance the appropriateness of the model for the expressions and culture of Mandarin speakers, the app collected voice data containing natural Mandarin spoken with a Taiwanese accent. The dataset contains 13,075 audio recordings submitted by 6,457 independent users and is 28,814 minutes in total. In the recordings, the participants talked freely and described their personal experiences and emotions. Three certified psychological counselors annotated all of the recordings using the same emotions used with RAVDESS. To process the subjectivity of the annotations, triangulation was adopted, and recordings with consistent annotations from at least two of the psychological counselors were retained. Ultimately, 1,961 training samples and 491 pen-and-paper test samples. The optimized Mandarin model has an accuracy rate of 79.8%. Note that due to the openness of the app, the dataset presents natural diversity but consequently lacks structured demographics, which prevents us from analyzing the performance of the model among certain ethnic groups." (see the Research instruments Section on lines 230-235 on Page 13 and 14, and lines 242-253 on Page 14 and 15)

Furthermore, to more clearly describe the data sorting and statistical analysis procedures, we have rewritten the Statistical analyses section. (see the Statistical analyses Section on lines 269-297 on Page 15-17)
