# Supplementary material for: Reliability, validity, and correlates of an AI voice emotion recognition app among nurses
Source: PLoS One. 2025 Dec 23;20(12):e0339365. doi: 10.1371/journal.pone.0339365 (PMC12725749; doi:10.1371/journal.pone.0339365)
Supplement: Attachment — Submitted filename: 20250803_Response Letter.docx [file pone.0339365.s005.docx]

***PLOS ONE***

Editor-in-Chief

Aug 3, 2025

Dear Editor-in-Chief,

Thank you very much for giving us the opportunity to again revise our paper entitled “Reliability and validity analysis of AI voice emotion recognition app and correlation between its results with the background variables and physical symptoms or diseases of nurses” (Manuscript ID PONE-D-25-20092R1). We have completed the required revisions, closely following the suggestions of the editor and reviewers. You may find the details of these revisions in the attached files. We believe that we have fulfilled all of the requirements for submission of the paper to *PLOS ONE*. If you find that any details have been overlooked, or if you need any further information, please do not hesitate to contact me.

Yours sincerely,

Wen-Pei Chang, RN, PhD

*PLOS ONE*

Referee Report on the Manuscript ID PONE-D-25-20092R1

“Reliability and validity analysis of AI voice emotion recognition app and correlation between its results with the background variables and physical symptoms or diseases of nurses”

We are grateful for your invaluable assistance in the revision of this paper. For this revision, we have closely followed the guidance provided by the reviewers.

Detailed responses to each of the reviewers’ comments have been provided below, with reviewers’ comments, followed by our comments and revisions.

In accordance with Reviewer #1’s suggestions:

The study addresses an important topic with potential practical value. However, the critical lack of detail regarding the AI app and its validation, coupled with limitations in study design and analysis, currently undermine the conclusions. Addressing these concerns, particularly providing full transparency about the AI tool and its validation, and controlling for confounders, is essential for the manuscript to make a significant scientific contribution. The ethical handling of sensitive voice data must also be clearly demonstrated.

***Response:***

We thank the reviewer for their affirmation of the importance of the research topic and its potential practical value as well as for their concrete and valuable suggestions. As shown below, we have made revisions and supplemented content as suggested:

1. Regarding the data processing details of the emotion recognition model

We have supplemented the following explanation in the Research instruments section: "To enhance the performance of the model and cultural appropriateness for Mandarin Chinese speakers, the app initially collected natural Mandarin Chinese voice data from individuals with a Taiwanese accent. The dataset contains Mandarin Chinese voice data from users in Taiwan. A total of 6,457 participants took part since data collection began, producing 13,075 items of voice data. The recordings reached 28,814 minutes in total length, averaging approximately 2.2 minutes each. Furthermore, over 100,000 voice files have been accumulated in the historical data of the platform, showing scalability potential for future model training. All voice data underwent standard preprocessing, including noise reduction and automatic segmentation, to ensure audio quality and analysis consistency. The emotions in each item of voice data were labeled by three nationally certified counseling psychologists. The emotion label classifications included eight basic emotions: worried, happy, sad, angry, surprised, fearful, calm, and neutral. Multi-label emotion classifications were permitted to reflect the emotional complexity of actual voice expressions. To increase label consistency and reliability, we employed the triangulation method and only included data in which at least two of the psychologists assigned the same labels. Ultimately, 13,075 recordings were included. Using an 8:1:1 ratio, the recordings were divided into a training set (*n* = 10,460), a validation set (*n* = 1,307), and a test set (*n* = 1,308). A total of 1,961 training samples and 491 test samples were retained. The optimized Mandarin Chinese model had a 79.8% accuracy rate. The open participation mechanism of the app allowed for a wide range of natural voice variations, thereby enhancing the ecological validity of the model and reflecting real-world usage scenarios. Note that we did not systematically obtain structured demographic statistics during the data collection process, which prevented us from analyzing model performance from an ethnic aspect. Future studies could include such variables for more comprehensive assessments of model versatility." (see the Research instruments Section on lines 219-242 on Page 13-14)

2. Regarding the training and validation of the emotion recognition model

We have supplemented the following explanation in the Research instruments section: "The emotion recognition model in this study was based on the wav2vec2-Large pre-trained audio representation framework and fine-tuned to adapt to Mandarin Chinese voice emotion recognition tasks. The model was trained using the triangulation-validated voice data. After each epoch, the performance of the model was validated using the test set, which was not part of the training, to observe the learning progress and generalization ability of the model. The model displayed a steady learning trend during the initial phases of training, with accuracy steadily rising from 48.9% after the first epoch to 85.9% after the fifteenth epoch. The relatively stable performance of the model demonstrated that voice emotion characteristics have been effectively learned with good classification performance and consistency." (see the Research instruments Section on lines 243-252 on Page 14)

3. Regarding the control of confounders

We have added a section with the heading “Explanatory model of emotion recognition app after controlling confounding factors” and Table 4 to the Results, with the following content: "To clarify the factors that impact the emotion recognition results from the app and control potential confounding factors, we referred to the background variables and psychological scale results presented in Tables 1 through 3 and employed multivariate statistical methods to control potential confounding effects. Confounders were selected based on the *p* values of the univariate analysis results to prevent over-tuning the model. The regression analysis results in Table 4 show that the model possesses significant explanatory power with regard to the recognition results of the various emotions (*R*^2^ ranging from .212 to .352, *p* < . 001). The VIFs of the variables were also all less than 2.5, thereby indicating the absence of multicollinearity. This analysis provides a basis for inference after controlling confounders and enhances the reliability and explanatory power of this study in terms of the validity of the emotion recognition model.

In the anger model, almost never exercising (β = .11, *p* = .025), the PANAS positive affect score (β = -.15, *p* = .007), the PSS score (β = - .13, *p* = .006), and the NAI score (β = .37, *p* < .001) were significant factors. In the fear model, peptic ulcers (β = .19, *p* < .001) and the PSS score (β = .36, *p* < .001) were significant factors. In the happiness model for the female participants, one or more cups of coffee a day (β = .11, *p* = .041), irregular menstrual cycles (β = -.13, *p* = .014), the PANAS positive affect score (β = .17, *p* = .006), the PANAS negative affect score (β = -.14, *p* = .034), and the BDI score (β = -.14, *p* = .034) were significantly correlated. In the sadness model for the female participants, irregular menstrual cycles (β = .30, *p* < .001), the PSS score (β = -.11, *p* = .031), the BDI score (β = .40, *p* < .001), and the NAI score (β = -.14, *p* = .003) were significantly correlated. Thus, these regression results indicate that after personal and psychological variables were controlled, the app still demonstrated significant validity and explanatory power in recognizing different emotions." (see the Results Section on lines 356-379 on Page 29-30 and Table 4 on Page 31-32)

Furthermore, to maintain the consistency of the entire paper, we have added the following content to the abstract: "To control for potential confounding variables, demographic and health-related factors including age, gender, work experience, exercise habits, and history of physical symptoms were collected and statistically adjusted in correlation analyses." and "Multiple linear regression analysis revealed the following: (1) nurses without regular exercise exhibited more anger, while those with peptic ulcers reported greater fear; (2) drinking over one cup of coffee daily was linked to higher happiness; and (3) female nurses with irregular menstrual cycles had lower happiness and greater sadness." (see the Abstract on lines 22-36 on Page 2-3)

In addition, we have supplemented the following explanation in the Discussion: "Multiple linear regression further confirmed that several background and psychological factors were significantly associated with the emotions detected by the app, supporting the preliminary psychological validity and explanatory power of the model." (see the Discussion Section on lines 406-408 on Page 36)

4. Regarding the ethical handling of sensitive voice data

We have added the following explanation to the Ethical considerations section: "Users themselves could remove their voice data on the platform at any time. Furthermore, all data were stored on a server encrypted by Microsoft Azure and managed in accordance with IRB-approved security regulations to ensure the security and legality of data access and storage processes." (see the Ethical considerations Section on lines 130-133 on Page 8)

In accordance with Reviewer #2’s suggestions:

Thank you for your revision that significantly enhanced the manuscript quality.

I recommend to accept the paper after my last comment: Regarding point 10 that I raised (Reviewer 2). I do not see any questionnaire that the nurses filled out even though you answered that you will provide it. Is this an error ? It should be in Supplementary file.

King regards.

***Response:***

We thank the reviewer for their affirmation of our revisions. We have supplemented and uploaded the questionnaire as a supplementary file. We apologize for this oversight and thank you for the reminder and correction.
