# Supplementary material for: Reliability, validity, and correlates of an AI voice emotion recognition app among nurses
Source: PLoS One. 2025 Dec 23;20(12):e0339365. doi: 10.1371/journal.pone.0339365 (PMC12725749; doi:10.1371/journal.pone.0339365)
Supplement: Attachment — Submitted filename: 20251101_Response Letter.docx [file pone.0339365.s006.docx]

***PLOS ONE***

Editor-in-Chief

Nov 2, 2025

Dear Editor-in-Chief,

Thank you very much for giving us the opportunity to again revise our paper entitled “Reliability, validity, and correlates of an AI voice emotion recognition app among nurses” (Manuscript ID PONE-D-25-20092R2). We have completed the required revisions, closely following the suggestions of the editor and reviewers. You may find the details of these revisions in the attached files. We believe that we have fulfilled all of the requirements for submission of the paper to *PLOS ONE*. If you find that any details have been overlooked, or if you need any further information, please do not hesitate to contact me.

Yours sincerely,

Wen-Pei Chang, RN, PhD

*PLOS ONE*

Referee Report on the Manuscript ID PONE-D-25-20092R2

“Reliability, validity, and correlates of an AI voice emotion recognition app among nurses”

In accordance with Editor’s suggestions:

1. The title is overly long; consider shortening to emphasize the primary focus.

***Response:***

We have shortened the title to make it more concise and focused on the main aim of the study. The revised title is as follows: “Reliability, validity, and correlates of an AI voice emotion recognition app among nurses”.

This version highlights the psychometric evaluation as the primary focus while retaining the key contextual information regarding its use among nurses. The change has been applied to both the title page and the manuscript.

2. Abstract lacks detail on the psychometric methods (e.g., type of reliability coefficients, what “moderate and positive correlation” means numerically).

***Response:***

We have revised the Methods and Results sections of the Abstract to include detailed psychometric information, specifying the type of reliability coefficient (intraclass correlation coefficient, ICC) and the numerical values of the convergent validity correlations (Page 2-3, Abstract, highlighted in red). Specifically, Pearson’s correlations between app-derived emotions and corresponding standardized scales were reported (anger–Novaco Anger Inventory-Short Form, *r* = .42; fear–Perceived Stress Scale, *r* = .41; happiness–Oxford Happiness Questionnaire, *r* = .45; sadness–Beck Depression Inventory-II, *r* = .47; all p < .001), and test-retest reliability was evaluated using ICCs (range = 0.73-0.80).

3. The AI system’s accuracy (≈70–80%) should be contextualized — was this sufficient for clinical applications?

***Response:***

We have clarified in the Discussion section that although the AI VER app achieved approximately 70% accuracy, which is acceptable for research and preliminary screening purposes, this is not sufficient for direct clinical decision-making (Page 28, Discussion, highlighted in red). The system should therefore be regarded as a supplementary tool to assist in identifying emotional tendencies rather than a diagnostic system.

4. Which validity do you mean? Criterion validity, convergent validity, or concurrent validity?

***Response:***

We have clarified that the validity examined in this study refers to convergent validity, as the AI VER emotion scores were correlated with standardized psychological scales assessing similar emotional constructs (e.g., anger, fear, happiness, and sadness) (Page 2, Abstract; Page 13, Statistical Analyses; highlighted in red).

5. Regarding sample size: Convenience sampling in one hospital limits generalizability; this needs to be discussed as a limitation
   ***Response:***

We agree with the reviewer’s concern and have addressed this issue in the Discussion section (Page 29, Limitations, highlighted in red). Specifically, we have acknowledged that participants were recruited from a single medical institution using convenience sampling, which limits the external validity and generalizability of the findings to other settings or populations. This statement has been retained and slightly refined to ensure clarity.

6. No factor analysis was performed to assess construct validity. And no inter-rater or internal consistency analysis for the app (e.g., Cronbach’s α or split-half reliability). Justify it

***Response:***

The AI VER app is an algorithmic model that classifies emotions from acoustic features rather than a multi-item psychometric scale. Therefore, traditional factor analysis for construct validity and internal consistency indices (e.g., Cronbach’s α or split-half reliability) were not applicable. Instead, psychometric evaluation focused on test-retest reliability and convergent validity to assess temporal stability and consistency with standardized psychological measures. This clarification has been added to the Discussion section (Page 26, Discussion, highlighted in red).

7. Effect sizes are small (e.g., β = .11 or .13) and their practical significance is not discussed.

***Response:***

We have added an explanation in the Discussion section to interpret the small effect sizes in a practical context (Page 25-26, Discussion, highlighted in red). Although some standardized regression coefficients (β = .11 or .13) were small, they still indicate meaningful patterns in nurses’ emotional states, reflecting subtle but consistent influences of lifestyle and health factors (e.g., exercise habits and menstrual irregularity) on emotion recognition outcomes. In large populations, such small effects may accumulate and have practical implications for nurse well-being and workplace interventions.

8. Discussion tends to overstate the implications — the conclusion that the app can “objectively detect emotional patterns” may be premature given moderate correlations and app accuracy.
   ***Response:***

We have revised both the Discussion and Conclusion sections to adopt a more cautious and evidence-based tone (Page 25-29, Discussion; Page 30, Conclusion; highlighted in red). Specifically, we have replaced the statement that the app can “objectively detect emotional patterns” with phrasing that emphasizes its preliminary and assistive role. The revised text now indicates that the AI VER app may tentatively reflect emotional tendencies and should be regarded as a supplementary or exploratory tool, rather than a diagnostic system, given its moderate correlations and 70-80% accuracy. These revisions clarify that the current findings provide preliminary evidence of potential utility and that further validation in larger and more diverse samples is required.

9. Causal language should be avoided (e.g., “Peptic ulcers were associated with fear”→ should be “Peptic ulcers correlated with fear”).

***Response:***

We have carefully revised the related sentences in the Discussion section to avoid any causal wording (Page 30, Discussion, highlighted in red). Specifically, we have replaced expressions implying causation with neutral, correlational terms (e.g., “were associated with”) and explicitly stated that the cross-sectional design limits causal inference.

10. How does the app perform compared with self-reported emotion scales (e.g., accuracy, AUC)?

***Response:***

The present study focused on psychometric validation (reliability and validity) rather than predictive performance evaluation. We have added clarification in the Discussion stating that metrics such as accuracy or AUC were not computed but that future studies may include ROC-AUC analyses with larger datasets to further quantify predictive capability (Page 26, Discussion, highlighted in red).

11. The limitation should include Single-institution sample bias: Self-report physical health data (potential recall bias).

***Response:***

The single-institution sampling bias had already been addressed in the Limitations section. We have now added a statement noting that physical health data (e.g., exercise habits and medical history) were self-reported and may be subject to recall bias or social desirability effects (Page 29, Limitations, highlighted in red).

12. Was there any independent validation to prevent bias from the company developing the app?

***Response:***

We have clarified this point in the revised Ethical considerations section (Page 6, Ethical considerations, highlighted in red). Although the VER model was developed and funded by Bamboo Technology Co., Ltd., all data collection, statistical analyses, and result interpretations were conducted independently by our academic research team at Taipei Medical University to ensure objectivity and prevent developer bias. The company was not involved in data analysis, research procedures, or study conclusions.
